# Scale and Safety: Analyzing the Association Between Intraoperative Difficulty and Achieving the Critical View of Safety in Laparoscopic Cholecystectomy

**DOI:** 10.7759/cureus.53408

**Published:** 2024-02-01

**Authors:** Hira Bakhtiar Khan, Ahmad Shiraz, Abdul Haseeb, Sana Hamayun, Aiman Ali, Muhammad Jawad Zahid, Qaidar Alizai, Maryam Karim, Sajid Ur Rehman, Irfan Ali

**Affiliations:** 1 General Surgery, Hayatabad Medical Complex Peshawar, Peshawar, PAK; 2 General Surgery, Rehman Medical Institute, Peshawar, PAK; 3 General Surgery, Mardan Medical Complex, Mardan, PAK

**Keywords:** critical view of safety, modified nassar scale, gallstone, bile duct injuries, laparoscopic cholecystectomy

## Abstract

Background: Laparoscopic cholecystectomy (LC) is the preferred method for gallstone removal, but bile duct injuries remain a concern. Achieving the critical view of safety (CVS) is pivotal in preventing such injuries. The aim of this study was to compare the rates of difficult LC in those with CVS achieved compared to those with CVS not achieved.

Methods: We performed a single-center prospective study on all patients with ultrasound-confirmed symptomatic gallstones. Patients were excluded if they refused to consent or if they underwent LC for indications other than gallstone disease. Patients were stratified into two groups as CVS not achieved and CVS achieved groups and compared for outcomes. Our primary outcome was the rate of intraoperative difficulty on the modified Nassar scale (MNS). Statistical analysis was performed using SPSS version 25.0 (IBM Corp., Armonk, NY).

Results: We included 70 patients who underwent LC for gallstones (CVS not achieved = 24 and CVS achieved = 46). The mean (SD) age was 42.2 (12.3) years, and 73.5% were females. The mean (SD) weight in our study cohort was 74.1 (10.9) kg, and there was no difference between the two groups in terms of the baseline demographic characteristics, disease characteristics, and comorbid conditions (p > 0.05). On univariate analyses, achieving CVS was associated with lower rates of higher-grade operative difficulty on the MNS and lower rates of length of stay of more than one day.

Conclusion: Achieving CVS is associated with easy LC based on significantly lower Nassar scores. These findings highlight the role of the MNS in the successful identification of the operative difficulty of LC and its correlation with achieving CVS.

## Introduction

Laparoscopic cholecystectomy (LC) is considered the gold standard treatment for symptomatic cholelithiasis with a high safety profile [1]. However, bile duct injuries (BDI) remain a major cause of morbidity in LC (0.08-0.3%) compared to open cholecystectomy (0.2%) [1,2]. The critical view of safety (CVS) is a technique devised to prevent such injuries, introduced by Strasberg in 1995 [2]. It involves three components: clearance of fat and other tissues from the Calot’s triangle, separating the gall bladder from the lower one-third of the cystic plate, and finally skeletonization of the cystic artery and duct [3,4].

Intraoperative injuries associated with LC are mainly the result of misidentification of anatomical structures usually due to severe inflammation or individual variations in the regional anatomy [5,6]. A classical cause of morbidity stems from injury to the common bile duct (CBD) secondary to misidentification of the CBD. Aberrant hepatic ducts have also been mistaken to be cystic ducts or cystic arteries, therefore giving rise to an increased incidence of BDI [4]. Such injuries lead to a prolonged postoperative disease course, reduced quality of life, and overall lower life expectancy [1].

The CVS is not to be applied in isolation but as an element of a comprehensive approach toward "Safe Cholecystectomy," which also constitutes good rescue strategies and clear access [2-7]. In cases where the CVS cannot be achieved, several bail-out strategies can be applied to reduce the risk of BDI, including the infundibular approach, subtotal cholecystectomy, and conversion to open cholecystectomy [4]. Several studies have been performed to reduce intraoperative errors in LC. The Nassar scale was introduced in 1995, and later modified in 1996, for grading the anatomy of the Calot’s triangle [8,9]. However, there is a lack of studies comparing the frequency of achieving successful CVS in easy vs. difficult LC. The aim of this study was to identify the frequency of achieving successful CVS in easy vs. difficult LCs using the modified Nassar scale (MNS).

## Materials and methods

We conducted a prospective observational study in a tertiary care hospital in a developing country over a period of six months from June 1, 2022, to December 31, 2022.

Inclusion and exclusion criteria

We included all adult (≥18 years) patients who underwent LC for cholelithiasis during the study period. Patients were excluded if they refused to consent and those who underwent LC for indications other than cholelithiasis.

Patient enrollment

After approval from the institutional review board, patients were identified based on the inclusion and exclusion criteria. Patients were approached and written informed consent was obtained followed by a chart review. Based on data from previous literature, we calculated a minimum sample size of 70 using the OpenEpi calculator for a 90% confidence interval and a 10% margin of error [10]. All patients were included using probability convenience sampling.

Patient stratification

We stratified our study sample into two groups based on the CVS score into CVS not achieved (score < 5) and CVS achieved (score = 5-9) and compared for outcomes.

Outcomes

Our primary outcome was the rate of difficult LC in those with CVS achieved vs. those with CVS not achieved. Difficult LC was defined as those who experience one or more of the following: conversion to open cholecystectomy, operative duration exceeding 60 minutes, vascular and/or biliary injuries, significant bleeding, and/or use of synthetic hemostats.

Data points

We collected data pertaining to patient demographic characteristics (age and gender), comorbid conditions, including diabetes mellitus, hypertension, and obesity (defined as body mass index ≥30 kg/m^2^), presenting signs and symptoms, concurrent pancreatitis, history of endoscopic cholangiopancreatography (ERCP), ultrasonographic characteristics, interventions performed, mortality, in-hospital complications, and hospital length of stay. We also collected data on laboratory findings, including total leukocyte count, serum bilirubin level, serum alanine transaminase (ALT) level, serum alkaline phosphatase (ALP), and serum C-reactive protein (CRP) level. We also abstracted data on a six-point ultrasonographic scoring chart and the CVS based on the MNS that uses intraoperative difficulty grade.

Statistical analyses

On univariate analyses, the study groups were compared in terms of baseline demographic and disease characteristics. Continuous normally distributed variables were reported as means and standard deviations (SD). Chi-square and Fisher's exact tests were performed for categorical variables. The two groups were compared in terms of outcomes using the chi-square test. A p-value of <0.05 was considered statistically significant. All analyses were conducted using SPSS version 25.0 (IBM Corp., Armonk, NY).

## Results

We enrolled a total of 70 patients in this study with a mean (SD) age of 42 (12.3) years. Of these, 73.5% were female, and the mean (SD) weight was 74 (10.9) kg. The most common presenting symptom was right upper quadrant pain (90%), followed by vomiting (40%) and fever (24%), and the most common sign on presentation was a positive Murphy sign (41%). In terms of symptom onset, 56% presented with acute symptoms, while 44% had chronic symptoms. The most common comorbid conditions included diabetes mellitus (16%), hypertension (30%), and ischemic heart disease (7%).

Among our study cohort, 52 patients experienced easy LC and 18 patients had difficult LC. There was no statistically significant difference in the demographic and baseline disease characteristics of these patients. However, the difficult LC group had significantly higher total bilirubin and higher serum CRP. Table 1 summarizes the baseline and disease characteristics of our study population.

**Table 1:** Comparison of baseline characteristics of patients with CVS achieved and those with CVS not achieved. ALP = alkaline phosphatase; ALT = alanine transaminase; CRP = C-reactive protein; CVS = critical view of safety; ERCP = endoscopic cholangiopancreatography; LC = laparoscopic cholecystectomy; n (%) = count (percentage); LOS = length of stay; RHC = right hypochondrium; SD = standard deviation, TLC = total leukocyte count. * = Bold p-values indicate statistical significance.

Variable	Total	CVS not achieved (n = 24)	CVS achieved (n = 46)	p-value*
Age, mean (SD)	42.2 (12.34)	43.6 (12.4)	41.5 (12.3)	0.609
Female, n (%)	50 (73.5)	16 (66.7)	34 (77.3)	0.343
Weight, mean (SD)	74.1 (10.94)	73.6 (10.5)	74.3 (11.2)	0.490
Comorbidities, n (%)				
Diabetes mellitus	10 (14.7)	1 (4.2)	9 (20.5)	0.070
Hypertension	20 (29.4)	2 (8.3)	18 (40.9)	0.005
Ischemic heart disease	5 (7.4)	1 (4.2)	4 (9.1)	0.457
Presenting signs and symptoms, n (%)				
RHC pain	61 (89.7)	20 (83.3)	41 (93.2)	0.202
Vomiting	27 (39.7)	10 (41.7)	17 (38.6)	0.807
Fever	17 (25.0)	9 (37.5)	8 (18.2)	0.079
Pancreatitis	16 (23.5)	3 (12.5)	13 (29.5)	0.113
ERCP history	3 (4.4)	2 (8.3)	1 (2.3)	0.245
Laboratory workup, mean (SD)				
TLC	9.45 (2.6)	9.7 (2.9)	9.2 (2.4)	0.481
Total bilirubin	0.7 (1.35)	1.2 (2.2)	0.5 (0.19)	<0.001
Serum ALT	60 (77.7)	62.1 (91.8)	58.6 (69.8)	0.674
Serum ALP	134.4 (78.27)	120.9 (50.0)	141.1 (89.7)	0.027
Serum CRP	2.57 (5.85)	4.2 (8.9)	1.6 (2.7)	0.006

On univariate analysis, a lower CVS score was associated with significantly higher grades of operative difficulty by Nassar score with no difference in the hospital length of stay (Table 2).

**Table 2:** Comparison of Nassar score and critical view of safety score. CVS = critical view of safety; LOS = length of stay. * = Bold p-values indicate statistical significance.

	CVS not achieved (n = 24)	CVS achieved (n = 46)	p-value
Nassar score			
Grade I-II	13 (54.2)	39 (88.6)	<0.001*
Grade III-V	11 (45.8)	5 (11.4)	
Hospital LOS of 1 day	12 (50.0)	17 (38.6)	0.365

## Discussion

The CVS was first coined by Strasberg and his colleagues in 1995 to minimize BDIs observed at an alarmingly high rate with the advent of LCs [3]. Today, it has become a popular tool in LCs conducted worldwide. Multiple studies have been conducted over time, which have proved the efficacy of CVS in reducing the morbidity and mortality previously associated with LCs [2,6,11].

Recently, Nassar et al. [7,12] introduced the MNS to grade the difficulty level faced intraoperatively during LC. Griffiths et al. [9] conducted a multi-center prospective study on a cohort of 8820 patients using the MNS to assess its accuracy in the operative field, thereby revealing a strong significance between a high MNS grade and adverse patient outcomes.

In our study, both the CVS score and MNS grading were applied to determine the efficacy of both scores in achieving a successful LC. A total of 68 patients were assessed to identify the level of significance between certain preoperative variables, a high MNS grade, and an inability to achieve a sufficient CVS score. It was observed that CVS was achievable in 83% of the patients with an MNS grade of 1-2 whereas with grade 3 or higher, the CVS score was unachievable in almost 68% of the patients. Similar results were obtained by Kar et al. [13] and Gupta et al. [14]. Increasing age and male gender were both reported to have a greater propensity toward high MNS difficulty grade [2,7], whereas little or no association was observed between disease onset and comorbidities.

While this study provides valuable insights into the frequency of achieving a CVS in LC, there are several limitations to consider. Firstly, this study was conducted at a single medical complex, which may limit the generalizability of the findings to a broader population. Additionally, the sample size of 70 patients, though calculated using appropriate statistical methods, may be considered relatively small for drawing definitive conclusions.

Future recommendations for this study include conducting larger, multi-center studies to validate the findings and increase generalizability. It is crucial to extend the follow-up period to assess long-term outcomes and complications associated with achieving a CVS. Investigating the influence of surgeon experience and training on CVS attainment is essential, along with exploring advanced imaging techniques like intraoperative ultrasound or cholangiography. Stratifying patients based on preoperative risk factors can help tailor surgical approaches, and implementing innovative techniques and technologies could optimize CVS achievement. Preoperative patient education and informed consent play a vital role in managing expectations. Quality improvement initiatives, including standardized approaches and regular training, can further enhance outcomes in LC.

## Conclusions

In conclusion, this study sheds light on the frequency of achieving CVS in LC. These findings suggest that achieving CVS is significantly associated with lower Nassar scores, indicating easier operative difficulty. This emphasizes the critical importance of meticulous surgical technique and preoperative assessment to enhance patient safety during cholecystectomy procedures.
